# The Association Between Gentrification, Health, and Social Cohesion of Older Adults

**DOI:** 10.21203/rs.3.rs-10778994/v1

**Published:** 2026-08-28

**Authors:** Dionne Bailey, Carrie Henning-Smith, Tetyana Shippee, David Haynes, Joseph E. Gaugler

**Affiliations:** 1 Department of Epidemiology and Public Health, University of Maryland, Baltimore, Baltimore, MD, USA; 2 Division of Health Policy & Management, University of Minnesota School of Public Health, Minneapolis, MN, USA; 3 Institute for Health Informatics, University of Minnesota, Minneapolis, MN, USA

**Keywords:** Gentrification, Neighborhoods, Health, Social Cohesion

## Abstract

Gentrification disproportionately affects older adults. The gentrification process may adversely affect older adults’ health and well-being, as well as that of other neighborhood residents, through cumulative stress and potential declines in social cohesion. Several studies have investigated the health effects of gentrification, but few have focused specifically on its impacts on health and/or social cohesion among underrepresented older adults at the national level. To address this gap, the study used 2011–2022/23 data from the National Health and Aging Trends Study (NHATS) and the American Community Survey (ACS) to examine the association between gentrification and self-reported health, mental health, and perceived social cohesion among older adults. Overall, in 2015, 4.5% of census tracts were classified as gentrified, and by 2022/23, this proportion had risen to 5.3%. We employed generalized linear models (GLMs) and difference-in-differences models for our results. The results showed an association between gentrification and poorer self-rated health (0.491, p < .05) and stronger perceived social cohesion (−0.910, p < .05) among older adults who resided in the same gentrified neighborhood over time, compared with those who remained in non-gentrified neighborhoods. Gentrification was not significantly associated with poorer mental health (1.186, p < .10). These results have implications for future public health research. These results suggest that as neighborhoods experience transformation, efforts should be made to include older adults in decision-making. It also remains essential to expand efforts to ascertain neighborhood factors that may impact the health and social context of older adults.

## INTRODUCTION

Living environments, including one’s home and surrounding community, are critically important to the well-being of older adults. Remaining in one’s home and familiar surroundings, or aging in place, can help protect older adults from potentially harmful psychological and physical health effects [1]. For older adults, familiarity with their surroundings promotes a sense of stability, comfort, and security. These qualities are crucial for reducing stress and anxiety [2]. Familiarity with surroundings can mitigate potential harmful health impacts. Most older adults aged 65 years or older (~77%) desire to age in place [3]. A community must strike a balance between older adults’ familiarity and their access to infrastructure, housing quality, and the resources they need to become “age-friendly” for successful aging in place [4].

Disruptions, like gentrification, could make aging in place more difficult. Ruth Glass first coined the term “gentrification,” defined as the process by which middle- and upper-income groups move into low-income urban neighborhoods, purchase and renovate properties, and thereby significantly alter the area’s social and economic landscape [5, p. 4]. Familiarity with one’s surroundings can help people maintain ongoing social engagement (such as being close to peers) and make good use of local services, both of which can affect health [6]. Gentrification may threaten older adults’ ability to maintain social ties and stay familiar with their surroundings. Maintaining social interaction is essential to aging in place because it supports older adults’ mental, physical, and emotional well-being [7]. Even so, shifting demographics in gentrifying communities may leave residents disoriented and unfamiliar, which could affect their health [11], with outcomes varying by individual-level factors such as income, race, and social support [8], [9].

According to Webber and colleagues (2023), views of home in older life are dynamic and alter over time because of gentrification-related neighborhood changes and the breakdown (or lack thereof) of social ties. Gentrification’s effects on neighborhood resources and social makeup put social cohesion at serious risk, potentially affecting residents’ health [11], [12]. Social cohesion is defined as unity and solidarity among residents, which confer numerous benefits on members, including increased social participation and improved well-being [13], [14]. Social interactions and place are significantly correlated [1], [10], and the place where older adults reside can impact their health [12], [15].

Many older adults view neighborhood transformation due to gentrification as detrimental to their quality of life and the networks to which they belong [16]. Although studies have considered the health effects of gentrification [17], [18], [19], very few have examined its implications for health and social cohesion among older adults at a national scale [20]. The objectives of this paper are to explore the association between gentrification and the self-rated health, mental health, and social cohesion of community-dwelling older adults over time.

## METHODS

### Study Design

A repeated cross-sectional study design was utilized, relying on data from 2011 to 2022/23 from the American Community Survey (ACS) and the National Health and Aging Trends Study (NHATS). The ACS and NHATS survey participants during comparable timeframes [21], [22]. The ACS is an extensive, continuous survey of the whole U.S. population, while the NHATS is a targeted, annual study focused exclusively on Medicare beneficiaries aged 65 and above. This research merged datasets using Federal Information Processing Standards (FIPS) codes, which serve as distinct geographic identifiers for counties across the U.S., thereby facilitating consistent linkage of county-level data over time.

The year 2011 was designated as the starting point for the creation of the gentrification variables included in this research. The year 2015 was chosen as the midpoint because it is relevant to the census data, and the NHATS survey items from this wave were comparable in structure and content to those from the 2022/23 wave. In 2015, 8,334 individuals participated in the NHATS, and in 2022/23 there were 6,327. The analysis included only those who completed both waves of data collection (n = 3,245) to ensure uniformity. Subsequently, the final sample was divided into two categories: those who had not relocated during the study period (n = 2,267) and those who moved from their 2015 residence (n = 922). Additionally, some participants (n = 3,082) were excluded from the analytical sample due to the dataset merger of rounds 5 and 12 because they did not complete both survey rounds.

### Variable Construction

#### Gentrification

To determine which areas are undergoing gentrification, methodologies established by Freeman (2005), Deng (2024), Ding and Hwang (2016), and Izenberg and colleagues (2018) were used. Census tracts were initially evaluated for eligibility based on socioeconomic criteria, utilizing the Census-Based Statistical Area (CBSA) framework to ensure geographic comparability. A tract is deemed eligible for gentrification if its median household income falls below the median income of its corresponding CBSA [26]. Among eligible tracts, gentrification status was assessed based on concurrent increases in four key indicators over subsequent years: median educational attainment (specifically, the percentage of residents with a college degree), median household income, median rent, and median housing prices. All variables must exceed the applicable CBSA median values for the same period for a tract to be designated as gentrified for this study.

#### Health

We assessed self-rated health (SRH) using an ordinal scale ranging from excellent to poor (1 = excellent; 5 = poor). The mental health assessment was conducted with the 2-item Patient Health Questionnaire (PHQ-2) and the 2-item Generalized Anxiety Disorder (GAD-2), both of which are established, concise screening tools for depression and anxiety, respectively. The PHQ-2 and GAD-2 have four inquiries, “Over the last month, how often have you: (a) had little interest or pleasure in doing things; (b) felt down, depressed, or hopeless; (c) felt nervous, anxious, or on edge; (d) been unable to stop or control worrying?” Response classifications encompass the following categories: “not at all,” “several days,” “more than half the days,” and “practically every day.” The PHQ-2 consists of items “a” and “b,” whereas the GAD-2 is made up of items “c” and “d. We examined values separately and combined them into a composite metric by totaling the individual variables. The scale ranges from excellent to poor, like the SRH measure. Previous studies have integrated the two for their evaluations and exhibited robust reliability and validity in assessing symptoms of both anxiety and depression [27], [28].

#### Social Cohesion

Social cohesion was assessed based on respondents’ answers to three NHATS survey questions: (1) “People in my community know each other well,” (2) “People are willing to help each other,” and (3) “People can be trusted.” Respondents self-defined their community or neighborhood in response to these questions. They answered each question using a three-point scale (1 = agree a lot, 2 = agree a little, 3 = do not agree). We combined them into an ordinal scale that ranges from 1 to 9. This scale ranges from low to high; a rating of “1” indicates high social cohesion, and a rating of “9” indicates low social cohesion. The measure of social cohesion was adapted from commonly cited literature [29], [30].

#### Covariates

The covariates of interest in the study were collected from NHATS. Sociodemographic variables were examined, encompassing age, race, gender, educational attainment, income, and marital status. Age was classified into five-year intervals (65–69, 70–74, 75–79, 80–84, 85–89, and 90+). Gender was classified as male or female. Race and ethnicity were categorized as White non-Hispanic, Black/African American non-Hispanic, Hispanic, and Other, owing to the limited sample size of Native Americans and Pacific Islanders. Age, gender, and race are key social determinants of health that can influence social and environmental factors [31]. Educational attainment was categorized into: less than high school, high school graduate, post-secondary education (associate’s degree), bachelor’s degree, and graduate degree. Educational attainment is a covariate because it influences various aspects of older adults’ lives, including cognitive performance, physical and mental health, and overall well-being [32], [33]. Marital status was categorized into five classifications: married/living with partner, separated, divorced, widowed, or never married. Marital status correlates with the general life satisfaction and well-being of older adults [34].

Census Divisions were classified into four regions: Northeast, Midwest, South, and West. The gentrification process is influenced by regional and local conditions and events [35]. Medicaid status was included as a covariate; respondents were asked whether they were enrolled in Medicaid (enrolled vs. not enrolled). Medicaid status in research frequently serves as a proxy indicator of socioeconomic status [36], [37]. It is essential to define the home- and community-based services (HCBS) available to those desiring to age in place, as it often serves as the primary or sole source of financing for long-term care provided outside institutional settings [38], [39]. The third covariate of interest pertains to housing, indicating whether the resident owns, rents, or has an alternative housing arrangement. These specified housing factors are essential in clarifying the consequences of gentrification [20].

Finally, we incorporated assessments of health and functional status. Initially, respondents were requested to answer “yes” or “no” about the presence of comorbidities, including heart attack, arthritis, osteoporosis, diabetes, lung disease, stroke, dementia, and cancer. The number of comorbidities was categorized into four groups: none, one, two, and three or more. Additionally, functional limitations were defined as a binary variable (yes/no) based on respondents’ responses to a series of questions assessing physical capabilities. Participants were specifically surveyed regarding their ability to execute the following tasks: walk six blocks, walk three blocks, ascend 20 stairs, ascend 10 stairs, carry 20 pounds, carry 10 pounds, kneel, bend, lift a heavy object overhead, reach overhead, open a sealed jar manually, and grasp small objects. Respondents answered each item with “yes” or “no.” Those who responded “yes” to at least one question regarding functional restrictions were categorized as having a functional limitation, while those who answered “no” to all were classed as having none. Particularly among older adults, comorbidity and functional limitations are closely associated with self-reported health status [40], [41].

### Statistical Analysis

Chi-square tests were used to compare the demographic characteristics of individuals living in gentrified and non-gentrified neighborhoods. We utilized a logistic regression model to evaluate the probability of change in older adults’ health and perceived social cohesion, while accounting for variables including gentrification, age, race/ethnicity, gender, income, living situation, census division, educational attainment, marital status, presence of comorbidities and/or functional limitations, and Medicaid coverage. A difference-in-differences methodology was used to evaluate temporal changes in health and perceived social cohesion associated with gentrification among residents who remained in place. The model compares differences in predicted probabilities of the health outcomes and perceived social cohesion before and after exposure to the gentrification process. Analytic weights produced by NHATS were employed to account for differing nonresponse rates and to estimate national prevalence estimates. All analyses were performed utilizing Stata within the University of Michigan enclave to access restricted data.

## RESULTS

Table 1 presents the characteristics of the study cohort by year, classified by residence in a gentrified or non-gentrified area (n = 3245). In 2015, 4.5% of census tracts were classified as gentrified; by 2022/23, this figure had increased to 5.3%. The average age range of participants in 2015 was 70–74 years, whereas in 2022/23, it shifted to 80–84 years. More than 50% of respondents identified as female in both gentrified and non-gentrified areas in 2015 and 2022/23. Of the individuals selected, 2267 (69.9%) continued to reside in the same place throughout the 7-year study period. Furthermore, income and rent differed significantly between gentrified and non-gentrified neighborhoods over time. From 2015 to 2022/23, median income increased from $40,000 to $42,000 in gentrified neighborhoods and from $30,000 to $33,051.50 in non-gentrified neighborhoods. Median rent also increased, from $882.05 to $937.50 and from $575.00 to $805.00, respectively.

### Logistic Regression Models

#### Self-Rated Health.

Table 2 illustrates the variation in self-rated health from 2015 to 2022/23. In the fully adjusted model, gentrification had no significant correlation with self-rated health in any year. Participants identifying as Non-Hispanic Black (2015 Coeff: 0.417, p < .001; 2022/23 Coeff: 0.213, p < .001) or Hispanic (2015 Coeff: 0.568, p < .001; 2022/23 Coeff: 0.275, p < .001) exhibited a higher propensity to report poorer self-rated health in both 2015 and 2022/23, in comparison to those identifying as Non-Hispanic White. Female respondents (2015 Coeff: −0.165, p<.001; 2022/23 Coeff: −0.185, p<.001), in contrast to their male counterparts, were less likely to indicate poorer self-rated health in both years 2015 and 2022/23. Education was strongly correlated with improved self-assessed health. Higher education was correlated with improved health outcomes. Individuals possessing a Master’s, Professional, or Doctoral degree indicated markedly superior self-rated health in both 2015 and 2022/23 (2015 Coefficient: −0.369, p < .001; 2022/23 Coefficient: −0.208, p < .01) relative to those with an education level below high school. Individuals with at least one comorbidity were more likely to indicate poorer self-rated health than those without any comorbidities. Functional limitations were significantly correlated with lower self-rated health (2015 Coeff: 0.438, p < .001; 2022/23 Coeff: 0.439, p < .001). Finally, Medicaid coverage, compared with its absence, was associated with poorer self-rated health (2015 Coeff: 0.213, p < .001; 2022/23 Coeff: 0.279, p < .001).

Additionally, Table 2 illustrates variations in self-rated health in 2022/23, categorized by whether people remained in the same neighborhood or moved. The findings indicated that Non-Hispanic Black (Coeff: 0.218, p < .001) and Hispanic (Coeff: 0.340, p < .001) respondents who did not relocate from their neighborhood had poorer self-rated health than their Non-Hispanic White counterparts who remained in the same neighborhood. Furthermore, female respondents (Remained Coeff: −0.192, p < .001; Moved Coeff: −0.197, p < .01) were less likely to report inferior self-rated health than their male counterparts. Individuals with a Master’s, Professional, or Doctoral degree had better self-rated health compared with those who never relocated (Coeff: −0.198, p < .01) and those who did relocate (Coeff: −0.246, p < .05).

#### Mental Health.

Table 3 illustrates changes in mental health from 2015 to 2022/23. Throughout the study period, individuals identifying as female showed a greater propensity for poorer mental health than their male counterparts (2015 Coeff: 0.186, p < .05; 2022/23 Coeff: 0.173, p < .05). Moreover, higher income was significantly and positively associated with mental health. In the years 2015 and 2022/23, respondents in income quartiles #3 (2015 Coeff: −0.578, p < .001; 2022/23 Coeff: −0.451, p < .01) and #4 (2015 Coeff: −0.701, p < .001; 2022/23 Coeff: −0.443, p < .05) exhibited more positive mental health relative to those in quartile #1 with lower income. The results indicated that those with poorer mental health were more likely to be renters compared to homeowners. In 2015 and 2022/23, renters (2015 Coeff: 0.235, p < .05; 2022/23 Coeff: 0.455, p < .001) had a higher propensity to report poorer mental health compared to homeowners. Furthermore, in both years, having two (2015 Coeff: 0.225, p < .05; 2022/23 Coeff: 0.501, p < .01) or three or more (2015 Coeff: 0.654, p < .001; 2022/23 Coeff: 1.121, p < .001) comorbidities, in contrast to respondents with none, correlated with poorer mental health. Ultimately, the presence of at least one functional limitation was associated with poorer mental health compared with individuals without such limitations (2015 Coeff: 0.714, p < .001; 2022/23 Coeff: 0.625, p < .001).

Additionally, Table 3 illustrates variations in mental health in 2022/23, categorized by whether people remained in the same neighborhood or moved. Upon further stratification, renters who remained in the same neighborhood (Coeff: 0.392, p < .01) and those who relocated (Coeff: 0.497, p < .05) exhibited poorer mental health compared to homeowners. Moreover, higher income correlated with improved mental health for both individuals who stayed in the same neighborhood and those who relocated. Respondents in income quartiles #3 (Coeff: −0.427, p < .05) and #4 (Coeff: −0.364, p < .05) showed a correlation with improved mental health relative to those in the lower-income quartile #1 among residents who stayed in the same neighborhood.

#### Social Cohesion.

In the fully adjusted model for perceived social cohesion (see Table 4), gentrification was not statistically significant in any year. Participants identifying as non-Hispanic Black (2015 Coeff: 0.263, p < .001; 2022/23 Coeff: 0.403, p < .001) exhibited a significant association with lower perceived social cohesion compared to those identifying as White in both 2015 and 2022/23. Moreover, participants identifying as Hispanic (2015 Coeff: 0.300, p < .05; 2022/23 Coeff: 0.140, p < .01) exhibited lower perceived social cohesion compared to those identifying as White in both 2015 and 2022/23. Specifically, when further categorized by whether residents remained in the same neighborhood or moved, non-Hispanic Black (Stayed Coeff: 0.363, p < .001; Remained Coeff: 0.441, p < .01) and Hispanic (Stayed Coeff: 0.473, p < .001; Moved Coeff: 0.921, p < .001) respondents exhibited a significant correlation with lower social cohesion relative to their White counterparts.

Individuals who identified as separated, divorced, or widowed had diminished levels of social cohesion relative to those who classified as married or living with a partner (2015 Coeff: 0.136, p < .05; 2022/23 Coeff: 0.137, p < .05). In both 2015 and 2022/23, older adults aged 85–89 (2015 Coeff: −0.391, p < .01; 2022/23 Coeff: −0.563, p < .001) exhibited greater levels of social cohesion compared to those aged 70 to 74. Furthermore, an analysis of these groups based on residency—those who remained in the same area versus those who relocated—revealed that the 85–89 age cohort (Remained Coeff: −0.596, p < .001; Moved Coeff: −0.497, p < .05) exhibited greater social cohesion than the 70–74 age group. In 2015, individuals in the third quartile of total income (Coeff: −0.356, p < .01) and the fourth quartile (Coeff: −0.322, p < .05) were more likely to have higher levels of social cohesion than those in the first quartile. In the year 2022/23, respondents with at least one functional limitation exhibited lower levels of social cohesion than those without any functional limitations (Coeff: 2.37, p < .01). Upon further analysis (see Table 4), individuals who either stayed in the same neighborhood or relocated exhibited a correlation of poorer social cohesion relative to those without functional limitations (Remained Coeff: 0.205, p < .05; Moved Coeff: 0.367, p < .05).

### Difference-in-Differences Models

The primary analysis of interest was a difference-in-differences model used to evaluate changes in self-rated health, mental health, and perceived social cohesion over time. In the fully adjusted models, the findings were largely stable. The coefficient for the difference-in-differences analysis of self-rated health was 0.491 (p < .05). This suggests that residents who remained in the same gentrified neighborhood experienced a deterioration in self-rated health over time compared with those residing in non-gentrified neighborhoods. In the fully adjusted mental health difference-in-differences model, the coefficient was 1.186 (p < .10), indicating that the mental health of residents who remained in gentrified neighborhoods did not differ significantly over time from that of residents in non-gentrified neighborhoods. Lastly, for perceived social cohesion, the coefficient in the difference-in-differences model was −0.910 (p < .05) for residents who stayed in the same gentrified neighborhood, compared with those in non-gentrified neighborhoods over time. This suggests a significant positive association with gentrification, indicating a stronger cohesive neighborhood over the 7 years.

## DISCUSSION

This present study assesses the association between gentrification, health, and the perceived social cohesion of older adults. The study extends the existing literature by demonstrating that, after adjustment for individual- and neighborhood-level covariates, gentrification was associated with poorer self-rated health among older adults who remained in gentrifying neighborhoods over time compared with those residing in neighborhoods that did not undergo gentrification. Conversely, gentrification was associated with increased perceived social cohesion, suggesting that neighborhood change may produce heterogeneous effects across different dimensions of health and social well-being. No statistically significant association was observed between gentrification and mental health among older adults who remained in gentrifying neighborhoods over the study period. Cross-sectional analyses further revealed persistent racial and ethnic disparities, with Non-Hispanic Black and Hispanic older adults reporting poorer self-rated health, poorer mental health, and lower perceived social cohesion than their Non-Hispanic White counterparts without the interaction of gentrification. Moreover, several sociodemographic and health-related characteristics, including age, household income, marital status, educational attainment, functional limitations, and comorbidity burden, were independently associated with the study outcomes.

Prior research revealed that neighborhoods experiencing gentrification frequently contain a substantial number of Black residents [42] as well as other minoritized and low-income populations [43]. This aligns with our results. The U.S. Census South Region, South Atlantic Division, had the highest percentage of residents living in gentrified neighborhoods (2015: 18.86%; 2022/23: 20.61%). This region has the highest percentage of minority residents, making up approximately 47% of its population [44]. Numerous studies on gentrification indicate that middle-class White residents and investors perceive racially diverse towns, particularly those with a Black population over 40%, as the most desirable for residential purchases and investments [16]. The Black population represents 14.7% of the total U.S. population [45].

Our results showed that income is significant in study outcomes, with higher income leading to better health and social cohesion. This implies that income can serve as a protective factor. Older non-Hispanic Black and Hispanic adults typically exhibit lower income levels and higher poverty rates relative to older non-Hispanic White adults [46]. In our study, over time, rent increased by 64%, whereas income increased for all residents by 10% in gentrified neighborhoods. The disparity between income and rent increases may create financial strain for residents of gentrified neighborhoods and exacerbate study outcomes. The financial strain on renters in gentrified areas may jeopardize the stability of lower-income households, leading to potential displacement. Residents in financial distress who relocate from gentrified neighborhoods due to rising costs may face greater financial constraints. They may face further financial difficulties arising from substantial declines in affordability as gentrification advances in other neighborhoods [25].

This paper is not without limitations. This study’s use of repeated cross-sectional studies prevents causal inference. Causal inference is relevant to research on the effects of neighborhoods on health [47]. There is no uniform method for measuring gentrification, given the multiple ways it has been defined and operationalized in the literature [48]. Discrepancies in quantifying gentrification can lead to nuanced interpretations of findings and limit attempts to synthesize its impacts in the current literature. Despite this limitation, we drew on the work of Freeman (2005), Izenberg and collaborators (2018), and Finio (2022) to conceptualize and analyze the gentrification process. Going forward, researchers should strive for a cohesive definition and standardized approach for gentrification. This will facilitate research that can be implemented with greater precision and compared with other studies. Another limitation of this study is the utilization of census tracts. Census tracts serve merely as a proxy for neighborhoods and do not necessarily align with the actual experiences and self-perceptions of older adults regarding their neighborhoods; they are also less effective in rural regions. Lastly, attrition may introduce bias. This bias occurs when participant numbers vary among groups and years, hence distorting the relationship between variables.

## CONCLUSION

In this study, the fully adjusted difference-in-differences analysis revealed a significant association between gentrification and poorer self-rated health, as well as stronger perceived social cohesion among older adults who remained in the same gentrified neighborhood over time, compared with those who remained in non-gentrified neighborhoods. Gentrification was not significantly associated with poorer mental health among older adults who remained in gentrified neighborhoods over time, compared with those who remained in non-gentrified neighborhoods.

Gentrification challenges healthy aging in urban environments and can exacerbate racial inequities at the neighborhood level. Appropriate policy interventions can mitigate these effects. Aging-in-place policies frequently recognize cultural diversity. However, implementation is infrequent, and structural disadvantage receives far less attention [50]. This oversight significantly hinders marginalized older adults from aging in place effectively. Policymakers should prioritize aligning policies across several domains to promote health equity.

## Figures and Tables

**Table 1.** Covariate Characteristics of Older Adults Living in Non-Gentrified and Gentrified Neighborhoods (*N* = 3245)

		2015 Non-Gentrified	2015 Gentrified		2022/23 Non-Gentrified	2022/23 Gentrified	
Variable	Category	%	%	P-value	%	%	P-value
Race/Ethnicity				0.836			0.913
	White, Non-Hispanic	83.39%	80.50%		83.37%	81.02%	
	Black, Non-Hispanic	7.55%	10.89%		7.54%	10.54%	
	Other (Native American, Asian, Hawaiian)	3.70%	2.13%		3.67%	2.82%	
	Hispanic	2.86%	3.75%		2.88%	3.32%	
	More than One	0.10%	0.00%		0.12%	0.00%	
	Don’t Know/Refused to Answer	2.40%	2.74%		2.42%	2.31%	
Age				0.000			0.000
	65–69	31.68%	1.96%		--	--	
	70–74	33.24%	38.17%		15.09%	0.00%	
	75–79	18.91%	25.75%		40.69%	31.15%	
	80–84	11.31%	19.78%		21.79%	29.46%	
	85–89	3.76%	9.75%		14.51%	21.46%	
	90+	1.10%	4.59%		7.92%	17.93%	
Gender				0.006			0.010
	Male	44.63%	33.78%		44.63%	35.56%	
	Female	55.37%	66.22%		55.37%	64.44%	
Income				0.016			0.022
	Quartile #1 (2015: $0-$16,799; 2022/23: $0-$19,999)	12.78%	20.14%		14.63%	24.00%	
	Quartile #2 (2015: $16,800-$32,861; 2022/23: $20,000-$39,999)	19.3%	29.46%		24.11%	27.91%	
	Quartile #3 (2015: $32,862-$63,499; 2022/23: $40,000-$77,999)	28.66%	28.58%		30.76%	25.99%	
	Quartile #4 (2015: $63,500+; 2022/23: $78,000+)	39.26%	21.82%		30.5%	21.10%	
Educational Attainment				0.000			0.003
	Less than High School	10.32%	23.42%		10.40%	19.91%	
	High School Graduate	24.29%	33.69%		24.54%	27.7%	
	Vocational, Technical, or Business, Some College, Associates	28.95%	28.34%		29.02%	27.28%	
	Bachelor’s Degree	17.70%	7.21%		17.62%	10.34%	
	Master's, Professional, or Doctoral	18.74%	7.35%		18.34%	14.77%	
Marital Status				0.016			0.004
	Married/Living with a Partner	62.12%	49.25%		51.91%	43.06%	
	Separated/Divorced/Widowed	34.67%	46.83%		44.92%	53.92%	
	Never Married	3.21%	3.93%		3.18%	3.02%	
Living Arrangement				0.009			0.021
	Own	81.9%	72.93%		73.47%	66.28%	
	Rent	12.71%	17.97%		18.28%	25.75%	
	Other Arrangement	5.38%	9.10%		8.25%	7.96%	
Count Comorbidities				0.005			0.057
	0	22.56%	15.47%		11.74%	8.31%	
	1	33.95%	27.86%		29.14%	25.71%	
	2	28.8%	38.71%		33.78%	33.45%	
	3+	14.68%	17.96%		25.35%	32.52%	
Functional Limitation				0.002			0.069
	No	47.07%	33.41%		28.58%	21.57%	
	Yes	52.93%	66.59%		71.42%	78.43%	
Medicaid Coverage				0.180			0.369
	Yes	7.61%	11.31%		10.32%	12.98%	
	No	92.39%	86.67%		89.68%	87.02%	
Census Division				0.929			0.970
	Northeast Region	18.45%	19.22%		18.05%	17.68%	
	Midwest Region	24.80%	27.92%		24.43%	28.41%	
	West Region	35.51%	26.86%		36.57%	30.4%	
	South Region	21.24%	26.01%		20.95%	23.5%	

Note: p-values compare residents who live in gentrified neighborhoods versus non-gentrified neighborhoods

**Table 2.** Self-Rated Health Regressions (Range: Excellent to Poor)

		2015	2022/23	2022/23 (Remained)	2022 /23 (Moved)
		*N=3245*	*N=3245*	*N=2267*	*N=992*
Variable	Category	Coefficient	Coefficient	Coefficient	Coefficient
Gentrified (Ref: Non-Gentrified)					
	Gentrified	0.141*	0.0443	0.024	0.118
Race (Ref: White Non-Hispanic)					
	Black Non-Hispanic	0.417***	0.213***	0.218***	0.157
	Hispanic	0.568***	0.275***	0.340***	0.088
	More than One	−0.783	0.11634	0.114	--
	Other	0.206*	0.067	0.1154	−0.135
	Don’t Know/Refused to Answer	0.023	0.006	−0.3775	0.290
Age		(Ref: 65–69)	(Ref: 70–74)		
	70–74	−0.0401	--	0.006	0.023
	75–79	−0.0448	0.00331	−0.0547	0.089
	80–84	−0.096	−0.029	0.004	0.097
	85–89	−0.0472	0.020	−0.059	−0.071
	90+	−0.067	−0.0653		
Gender (Ref: Male)				−0.192***	−0.197**
	Female	−0.165***	−0.185***		
Marital Status (Ref: Married/Living with Partner)				0.029	−0.090
	Separated/Divorced/Widowed	−0.117**	−0.008	0.181	−0.197
	Never Married	0.044	0.070		
Income (Ref: Quartile #1)				−0.0067	0.089
	Quartile #2	−0.056	0.0098	−0.055	−0.238
	Quartile #3	−0.171**	−0.119*	−0.107	−0.105
	Quartile #4	−0.264***	−0.137*		
Living Arrangement (Ref: Own)				0.106	0.050
	Rent	0.093*	0.037	0.040	0.138
	Other Arrangement	0.145*	0.057		
Education (Ref: Less than High School)				−0.020	−0.049
	High School Graduate	−0.141*	−0.034	−0.102	−0.199*
	Vocational, Technical, or Business, Some College, Associates	−0.189**	−0.122*	−0.044	−0.215
	Bachelor’s Degree	−0.253**	−0.087	−0.198**	−0.246*
	Master's, Professional, or Doctoral	−0.369***	−0.208**		
Count Comorbidities (Reference: Zero)				0.228***	0.184
	1	0.271***	0.216***	0.445***	0.412**
	2	0.444***	0.436***	0.786***	0.572***
	3+	0.757***	0.713***		
Functional Limitation (Ref: No)				0.490***	0.534***
	Yes	0.438***	0.493***		
Medicaid Coverage (Ref: No)				0.234***	0.402***
	Yes	0.213***	0.279***		
Census Division (Ref: Northeast Region)				0.074	−0.032
	Midwest Region	0.032	0.040	0.086	−0.005
	South Region	0.043	0.056	−0.019	0.030
	West Region	−0.041	0.000		

Note: Negative values indicate better self-rated health. Positive values indicate poorer self-rated health.

*** *p* < .001,

** *p* < .01,

* *p* < .05.

**Table 3.** Mental Health Regressions (Range: High to Low)

		2015	2022/23	2022/23 (Remained)	2022/23 (Moved)
		*N=3245*	*N=3245*	*N=2267*	*N=992*
Variable	Category	Coefficient	Coefficient	Coefficient	Coefficient
Gentrified (Ref: Non-Gentrified)					
	Gentrified	−0.003	−0.048	0.002	−0.042
Race (Ref: White Non-Hispanic)					
	Black Non-Hispanic	0.082	−0.124	−0.091	−0.229
	Other	0.252	−0.096	−0.394	0.330
	Hispanic	0.240	0.339	0.342	0.349
	More than One	−0.530	−0.925	−0.875	
	Don’t Know/Refused to Answer	0.279	0.020	1.040	−1.253
Age		(Ref: 65–69)	(Ref: 70–74)		
	70–74	−0.121	–	0.266	−0.059
	75–79	−0.177	. 2050919	0.181	−0.126
	80–84	−0.176	0.088	0.118	−0.222
	85–89	−0.289	0.048	0.312	0.001
	90+	−0.180	0.284		
Gender (Ref: Male)				0.115	0.223
	Female	0.186*	0.173*		
Marital Status (Ref: Married/Living with Partner)				−0.088	−0.433*
	Separated/Divorced/Widowed	−0.144	−0.217*	−0.100	−0.492
	Never Married	−0.106	−0.260		
Income (Ref: Quartile #1)				−0.197	0.145
	Quartile #2	−0.386**	−0.105	−0.427**	−0.440
	Quartile #3	−0.578***	−0.451**	−0.364*	−0.477
	Quartile #4	−0.701***	−0.443*		
Living Arrangement (Ref: Own)				0.392**	0.497*
	Rent	0.253**	0.455***	−0.210	−0.09
	Other Arrangement	0.252	−0.106		
Education (Ref: Less than High School)				−0.172	−0.153
	High School Graduate	−0.254	−0.189	−0.303*	−0.497
	Vocational, Technical, or Business, Some College, Associates	−0.306	−0.336**	−0.497**	−0.428
	Bachelor’s Degree	−0.418*	−0.474**	−0.570**	−0.432
	Master's, Professional, or Doctoral	−0.215	−0.500**		
Count Comorbidities (Reference: Zero)				0.301	0.049
	1	0.001	0.235	0.570***	0.307
	2	0.225*	0.501**	1.204***	0.937**
	3+	0.654***	1.121***		
Functional Limitation (Ref: No)				0.692***	0.522*
	Yes	0.714***	0.625***		
Medicaid Coverage (Ref: No)				0.314*	0.162
	Yes	0.485***	0.256		
Census Division (Ref: Northeast Region)				−0.383	0.243
	Midwest Region	−0.056	−0.222	−0.120	0.486**
	South Region	0.020	0.036	−0.204	0.224
	West Region	0.179	−0.101		

Note: Negative values indicate better self-rated health. Positive values indicate poorer self-rated health.

*** *p* < .001,

** *p* < .01,

* *p* < .05.

**Table 4.** Social Cohesion Regressions (Range: High to Low)

		2015	2022/23	2022/23 (Remained)	2022/23 (Moved)
		*N=3245*	*N=3245*	*N=2267*	*N=992*
Variable	Category	Coefficient	Coefficient	Coefficient	Coefficient
Gentrified (Ref: Non-Gentrified)					
	Gentrified	0.078	0.063	0.066	0.042
Race (Ref: White Non-Hispanic)					
	Black Non-Hispanic	0.263***	0.403***	0.363***	0.441**
	Other	−0.100	0.115	0.090	0.306
	Hispanic	0.300*	0.594***	0.473***	0.921***
	More than One	−0.509	−1.455	−1.496	--
	Don’t Know/Refused to Answer	0.417	−0.210	−0.494	−0.246
Age		(Ref: 65–69)	(Ref: 70–74)		
	70–74	−0.040	–	−0.189	−0.069
	75–79	−0.277	−0.147	−0.448***	−0.443
	80–84	−0.144	−0.434***	−0.596***	−0.497*
	85–89	−0.391**	−0.563***	−0.387**	−0.472
	90+	−0.269	−0.429***		
Gender (Ref: Male)				−0.155**	−0.168
	Female	−0.123	−0.176**		
Marital Status (Ref: Married/Living with Partner)				0.109	0.120
	Separated/Divorced/Widowed	0.136*	0.137*	0.306	−0.678*
	Never Married	0.143	0.046		
Income (Ref: Quartile #1)				0.141	−0.144
	Quartile #2	−0.231*	0.014	−0.222	−0.506*
	Quartile #3	−0.356**	−0.330**	−0.148	−0.374
	Quartile #4	−0.322*	−0.231		
Living Arrangement (Ref: Own)				0.369***	0.173
	Rent	0.066	0.174**	−0.163	0.221
	Other Arrangement	−0.036*	−0.039		
Education (Ref: Less than High School)				0.133	−0.252
	High School Graduate	−0.254	0.036	0.075	−0.301
	Vocational, Technical, or Business, Some College, Associates	−0.201	−0.030	−0.078	−0.307
	Bachelor’s Degree	−0.222	−0.136	0.129	−0.409*
	Master's, Professional, or Doctoral	−0.205	−0.031		
Count Comorbidities (Reference: Zero)				0.105	0.113
	1	−0.009	0.121	0.198	0.131
	2	0.034	0.185	0.317	0.197
	3+	0.215	0.291**		
Functional Limitation (Ref: No)				0.205*	0.367*
	Yes	0.101	0.237**		
State Medicaid (Ref: No)				0.080	−0.091
	Yes	0.003	0.037		
Census Division (Ref: Northeast Region)				−0.047	−0.300
	Midwest Region	0.031	−0.131	0.004	0.020
	South Region	0.059	−0.013	−0.031	0.038
	West Region	0.207	−0.017		

Note: Negative values indicate higher social cohesion. Positive values indicate lower social cohesion.

*** *p* < .001,

** *p* < .01,

* *p* < .05.

## References

[1] Ratnayake M., Lukas S., Brathwaite S., Neave J., and Henry H., “Aging in Place:,” Del. J. Public Health, vol. 8, no. 3, pp. 28–31, Aug. 2022, doi: 10.32481/djph.2022.08.007. PMC9495472 36177171

[2] Meagher B. R. and Cheadle A. D., “Distant from others, but close to home: The relationship between home attachment and mental health during COVID-19,” J. Environ. Psychol., vol. 72, p. 101516, Dec. 2020, doi: 10.1016/j.jenvp.2020.101516. 36540649 PMC9756114

[3] AARP, “2021 AARP Home and Community Preferences Survey,” AARP. Accessed: Nov. 11, 2024. [Online]. Available: https://www.aarp.org/pri/topics/livable-communities/housing/2021-home-community-preferences/

[4] Choi Y. J., “Understanding Aging in Place: Home and Community Features, Perceived Age-Friendliness of Community, and Intention Toward Aging in Place,” The Gerontologist, vol. 62, no. 1, pp. 46–55, May 2021, doi: 10.1093/geront/gnab070. PMC8759482 34043782

[5] Lees L., Slater T., and Wyly E., Gentrification. 2013.

[6] Dye C. J., Willoughby D. F., and Battisto D. G., “Advice from Rural Elders: What it Takes to Age in Place,” Educ. Gerontol., vol. 37, no. 1, pp. 74–93, Dec. 2010, doi: 10.1080/03601277.2010.515889.

[7] Pinazo-Hernandis S., Blanco-Molina M., and Ortega-Moreno R., “Aging in Place: Connections, Relationships, Social Participation and Social Support in the Face of Crisis Situations,” Int. J. Environ. Res. Public. Health, vol. 19, no. 24, p. 16623, Dec. 2022, doi: 10.3390/ijerph192416623. 36554504 PMC9779458

[8] Crewe S. E., “Aging and Gentrification: The Urban Experience,” Urban Soc. Work, vol. 1, no. 1, pp. 53–64, Jan. 2017, doi: 10.1891/2474-8684.1.1.53.

[9] Wilder V., Mirto A.-L., and Arniella G., “The Health Impact of Gentrification,” vol. 2, no. 5, 2017.

[10] Webber R., May V., and Lewis C., “Ageing in Place Over Time: The Making and Unmaking of Home.” Accessed: Jul. 30, 2025. [Online]. Available: https://journals-sagepub-com.ezp3.lib.umn.edu/doi/10.1177/13607804221089351

[11] Berkman L. F. and Kawachi I., Social Epidemiology. Oxford University Press, 2000.

[12] Hernandez L. M., Blazer D. G., and B. Institute of Medicine (US) Committee on Assessing Interactions Among Social, “The Impact of Social and Cultural Environment on Health,” in Genes, Behavior, and the Social Environment: Moving Beyond the Nature/Nurture Debate, National Academies Press (US), 2006. Accessed: Jul. 30, 2025. [Online]. Available: https://www.ncbi.nlm.nih.gov/sites/books/NBK19924/20669442

[13] Keith J., Old people, new lives: community creation in a retirement residence / Jennie-Keith Ross. Chicago: University of Chicago Press, 1977.

[14] Streib G. F. and Metsch L. R., “Conflict in retirement communities: Applying an analytical framework,” Res. Aging, vol. 24, no. 1, pp. 67–86, 2002, doi: 10.1177/0164027503024001005.

[15] Kawachi I., Kennedy B. P., Lochner K., and Prothrow-Stith D., “Social capital, income inequality, and mortality,” Am. J. Public Health, vol. 87, no. 9, pp. 1491–1498, Sep. 1997, doi: 10.2105/ajph.87.9.1491. 9314802 PMC1380975

[16] Hwang J. and Sampson R. J., “Divergent Pathways of Gentrification: Racial Inequality and the Social Order of Renewal in Chicago Neighborhoods,” Am. Sociol. Rev., vol. 79, no. 4, pp. 726–751, Aug. 2014, doi: 10.1177/0003122414535774.

[17] Gibbons J., Barton M., and Brault E., “Evaluating gentrification’s relation to neighborhood and city health,” PLOS ONE, vol. 13, no. 11, p. e0207432, Nov. 2018, doi: 10.1371/journal.pone.0207432. 30452460 PMC6242354

[18] Tran L. D., Rice T. H., Ong P. M., Banerjee S., Liou J., and Ponce N. A., “Impact of gentrification on adult mental health,” Health Serv. Res., vol. 55, no. 3, pp. 432–444, Jun. 2020, doi: 10.1111/1475-6773.13264. 31957022 PMC7240775

[19] Versey H. S., “Gentrification, Health, and Intermediate Pathways: How Distinct Inequality Mechanisms Impact Health Disparities,” Hous. Policy Debate, vol. 33, no. 1, pp. 6–29, Jan. 2023, doi: 10.1080/10511482.2022.2123249.

[20] Schnake-Mahl A. S., Jahn J. L., Subramanian S. V., Waters M. C., and Arcaya M., “Gentrification, Neighborhood Change, and Population Health: a Systematic Review,” J. Urban Health Bull. N. Y. Acad. Med., vol. 97, no. 1, pp. 1–25, Feb. 2020, doi: 10.1007/s11524-019-00400-1. PMC7010901 31938975

[21] Freedman V. A. and Kasper J. D., “Cohort Profile: The National Health and Aging Trends Study (NHATS),” Int. J. Epidemiol., vol. 48, no. 4, pp. 1044–1045g, Aug. 2019, doi: 10.1093/ije/dyz109. 31237935 PMC6934030

[22] Herman E., “The American Community Survey: An introduction to the basics,” Gov. Inf. Q., vol. 25, no. 3, pp. 504–519, Jul. 2008, doi: 10.1016/j.giq.2007.08.006.

[23] Freeman L., “Displacement or Succession?: Residential Mobility in Gentrifying Neighborhoods,” Urban Aff. Rev., vol. 40, no. 4, pp. 463–491, Mar. 2005, doi: 10.1177/1078087404273341.

[24] Deng H. H., “Shifting Landscapes: The Effects of Gentrification on Homelessness in Los Angeles County,” 2024.

[25] Ding L. and Hwang J., “The Consequences of Gentrification: A Focus on Residents’ Financial Health in Philadelphia,” Federal Reserve Bank of Philadelphia, Working paper (Federal Reserve Bank of Philadelphia) 16–22, Jul. 2016. doi: 10.21799/frbp.wp.2016.22.

[26] Izenberg J. M., Mujahid M. S., and Yen I. H., “Health in changing neighborhoods: A study of the relationship between gentrification and self-rated health in the state of California,” Health Place, vol. 52, pp. 188–195, Jul. 2018, doi: 10.1016/j.healthplace.2018.06.002. 29957396 PMC6181134

[27] Kroenke K., Spitzer R. L., and Williams J. B. W., “The PHQ-9,” J. Gen. Intern. Med., vol. 16, no. 9, pp. 606–613, Sep. 2001, doi: 10.1046/j.1525-1497.2001.016009606.x. 11556941 PMC1495268

[28] Mukuria C., Franklin M., and Hinde S., “Mapping functions for the PHQ-9 and GAD-7 to generate EQ-5D-3L for economic evaluation,” Eur. J. Health Econ., vol. 26, no. 1, pp. 63–70, 2025, doi: 10.1007/s10198-024-01692-0. 38664317 PMC11743390

[29] Kasper J. D. and Freedman V. A., “National Health and Aging Trends Study: User guide (Rounds 1 & 2, Final release),” 2014, [Online]. Available: www.nhats.org

[30] Latham K. and Clarke P. J., “Neighborhood Disorder, Perceived Social Cohesion, and Social Participation Among Older Americans: Findings From the National Health & Aging Trends Study,” J. Aging Health, vol. 30, no. 1, pp. 3–26, Jan. 2018, doi: 10.1177/0898264316665933. 27566464

[31] E. National Academies of Sciences , “Social Determinants of Health and Health Equity,” in The Future of Nursing 2020–2030: Charting a Path to Achieve Health Equity, National Academies Press (US), 2021. Accessed: Aug. 21, 2025. [Online]. Available: https://www.ncbi.nlm.nih.gov/books/NBK573923/34524769

[32] Adams S. J., “Educational Attainment and Health: Evidence from a Sample of Older Adults,” Educ. Econ., vol. 10, no. 1, pp. 97–109, Apr. 2002, doi: 10.1080/09645290110110227.

[33] Leggett A., Clarke P., Zivin K., McCammon R. J., Elliott M. R., and Langa K. M., “Recent Improvements in Cognitive Functioning Among Older U.S. Adults: How Much Does Increasing Educational Attainment Explain?,” J. Gerontol. Ser. B, vol. 74, no. 3, pp. 536–545, Feb. 2019, doi: 10.1093/geronb/gbw210. PMC6377030 28329815

[34] Gove W. R., Hughes M., and Style C. B., “Does Marriage Have Positive Effects on the Psychological Well-Being of the Individual?,” J. Health Soc. Behav., vol. 24, no. 2, pp. 122–131, 1983, doi: 10.2307/2136639. 6886367

[35] Shaw K., “Gentrification: What It Is, Why It Is, and What Can Be Done about It - Shaw - 2008 - Geography Compass - Wiley Online Library.” Accessed: Jul. 30, 2025. [Online]. Available: https://compass.onlinelibrary.wiley.com/doi/abs/10.1111/j.1749-8198.2008.00156.x?casa_token=−3F5UxSkzdgAAAAA:ys0TxOPpsBf55neaO_h-rEx3ji1iBCAT9w7XGrDvGkaHcQDLLqtqdQwrcNuA0isX3UmDSivKsQGLYs8

[36] Marcin J. P., Schembri M. S., He J., and Romano P. S., “A Population-Based Analysis of Socioeconomic Status and Insurance Status and Their Relationship With Pediatric Trauma Hospitalization and Mortality Rates,” Am. J. Public Health, vol. 93, no. 3, pp. 461–466, Mar. 2003, doi: 10.2105/ajph.93.3.461. 12604496 PMC1447764

[37] Yuma-Guerrero P., Orsi R., Lee P.-T., and Cubbin C., “A systematic review of socioeconomic status measurement in 13 years of U.S. injury research,” J. Safety Res., vol. 64, pp. 55–72, Feb. 2018, doi: 10.1016/j.jsr.2017.12.017. 29636170 PMC10372816

[38] MACPAC, “Home- and community-based services,” MACPAC. Accessed: Aug. 21, 2025. [Online]. Available: https://www.macpac.gov/subtopic/home-and-community-based-services/

[39] O’Keeffe J. , “Understanding Medicaid Home and Community Services: A Primer, 2010 Edition,” ASPE. Accessed: Aug. 21, 2025. [Online]. Available: http://aspe.hhs.gov/reports/understanding-medicaid-home-community-services-primer-2010-edition-0

[40] Koroukian S. M. , “Combinations of Chronic Conditions, Functional Limitations, and Geriatric Syndromes that Predict Health Outcomes,” J. Gen. Intern. Med., vol. 31, no. 6, pp. 630–637, Jun. 2016, doi: 10.1007/s11606-016-3590-9. 26902246 PMC4870414

[41] Williams J. S. and Egede L. E., “The Association Between Multimorbidity and Quality of Life, Health Status and Functional Disability,” Am. J. Med. Sci., vol. 352, no. 1, pp. 45–52, Jul. 2016, doi: 10.1016/j.amjms.2016.03.004. 27432034

[42] Croff R., Hedmann M., and Barnes L. L., “Whitest City in America: A Smaller Black Community’s Experience of Gentrification, Displacement, and Aging in Place,” The Gerontologist, vol. 61, no. 8, pp. 1254–1265, Mar. 2021, doi: 10.1093/geront/gnab041. 33772304 PMC8599195

[43] Hwang J. and Ding L., “Unequal Displacement: Gentrification, Racial Stratification, and Residential Destinations in Philadelphia,” Am. J. Sociol., vol. 126, no. 2, pp. 354–406, Sep. 2020, doi: 10.1086/711015.

[44] U.S. Census Bureau, “Census profile: South Atlantic Division,” American Community Survey 1-year estimates. Accessed: Aug. 06, 2026. [Online]. Available: http://censusreporter.org/profiles/03000US5-south-atlantic-division/

[45] M G.. and Passel J. S., “Facts About the U.S. Black Population,” Pew Research Center. Accessed: Jun. 30, 2025. [Online]. Available: https://www.pewresearch.org/race-and-ethnicity/fact-sheet/facts-about-the-us-black-population/

[46] Ochieng N., Cubanski J., Neuman T., Artiga S., and Damico A., “Racial and Ethnic Health Inequities and Medicare - Education, Poverty, and Wealth - 9642,” KFF. Accessed: Aug. 07, 2025. [Online]. Available: https://www.kff.org/report-section/racial-and-ethnic-health-inequities-and-medicare-education-poverty-and-wealth/

[47] Diez Roux A. V. and Mair C., “Neighborhoods and health,” Ann. N. Y. Acad. Sci., vol. 1186, pp. 125–145, Feb. 2010, doi: 10.1111/j.1749-6632.2009.05333.x. 20201871

[48] Firth C. L., Fuller D., Wasfi R., Kestens Y., and Winters M., “Causally speaking: Challenges in measuring gentrification for population health research in the United States and Canada,” Health Place, vol. 63, p. 102350, May 2020, doi: 10.1016/j.healthplace.2020.102350. 32543436

[49] Finio N., “Measurement and Definition of Gentrification in Urban Studies and Planning,” J. Plan. Lit., vol. 37, no. 2, pp. 249–264, May 2022, doi: 10.1177/08854122211051603.

[50] Finlay J. M., McCarron H. R., Statz T. L., and Zmora R., “A Critical Approach to Aging in Place: A Case Study Comparison of Personal and Professional Perspectives from the Minneapolis Metropolitan Area,” J. Aging Soc. Policy, vol. 33, no. 3, pp. 222–246, May 2021, doi: 10.1080/08959420.2019.1704133. 31856684

